# CRISPR/Cas9-based modulation of *V-PPase* expression in rice improves grain quality and yield under high nighttime temperature

**DOI:** 10.1007/s00299-025-03504-y

**Published:** 2025-05-10

**Authors:** Flávia Barbosa Silva Botelho, Soumen Nandy, Vibha Srivastava

**Affiliations:** 1 Department of Crop, Soil and Environmental Sciences, University of Arkansas System Division of Agriculture, Fayetteville, AR USA; 2 Agriculture Department, Federal University of Lavras, Lavras, Brazil

## Abstract

Transcriptional modulation of the vacuolar H^+^ translocating pyrophosphatase expressed specifically in the endosperm and reproductive tissue of rice improves its spikelet fertility and reduces grain chalkiness under high nighttime temperature.

**Supplementary Information:**

The online version contains supplementary material available at 10.1007/s00299-025-03504-y.

Warming trend of the climate has been linked with yield losses in many crops. Like other cereals, rice is highly sensitive to above-typical temperatures during reproductive and grain filling stages. In fact, nighttime temperatures have risen faster than daytime temperatures in many parts of the world. For this reason, rice studies in recent years have focused on the effect of high nighttime temperature (HNT) on grain yield. Several studies have shown that HNT disturbs key processes in reproductive development and grain filling that lead to reduced spikelet fertility (SF) and enhanced grain chalkiness (Srivastava et al. 2024). Chalkiness is the opaque area on the grain, and it is not just an appearance issue, it also impacts milling quality. Above the generally acceptable chalk values (6–10%), every 1% increase in chalkiness leads to 1% decline in head rice yield (HRY) (Zhao and Fitzgerald 2013). Therefore, breeding HNT tolerance is vital for safeguarding grain yields from heat waves in the future. However, breeding efforts have been impeded by the lack of reliable tolerance alleles in modern cultivars. Breeding is also complicated by the complex nature of HNT tolerance as not only SF and grain quality traits, but other yield components, such as panicle length, grain width, grain size, and grain weight, are also affected by HNT in a genotype-dependent manner. Not surprising, hundreds of QTLs have been identified in the genomics studies. Of which, *Chalk5* (Os05g0156900) is most notable as it stands as one of the few functionally validated QTL (Fan et al. 2024; Gann et al. 2023; Li et al. 2014).

*Chalk5* was identified in chalky *indica* rice. It encodes a vacuolar H^+^ translocating pyrophosphatase (V-PPase) that is most strongly expressed in the reproductive tissue and developing caryopses (Li et al. 2014). V-PPase regulates metabolic activities by maintaining cellular pH and preventing the buildup of inorganic pyrophosphate (PPi). Li et al. (2014) found that hyperactivity of *Chalk5* in young caryopses contributes to chalkiness in mature grains, and this hyperactivity is based on promoter elements found in *indica* rice that develop chalky kernels. These promoter elements are absent in the *V-PPase* allele of non-chalky rice, referred to as *VPP5* to distinguish it from *Chalk5* associated with chalky trait. However, transcriptional modulation of *VPP5* in non-chalky rice, Nipponbare, by CRISPR/Cas9-based mutagenesis of its promoter led to substantial reduction in grain chalkiness under HNT (Gann et al. 2023). This was very encouraging, because most modern cultivars succumb to undesirable levels of chalkiness under HNT that is a major threat to rice production (Srivastava et al. 2024). Here, we determined whether the CRISPR-based *vpp5* allele is a reliable source of HNT tolerance by (a) analyzing yield traits of the Nipponbare line harboring *vpp5* allele (Nip_vpp5), (b) validating the effect of *VPP5* promoter mutagenesis in Kitaake rice through CRISPR/Cas9, and (c) exploring the underlying mechanism by RNA-seq.

For phenotypic analysis, plants were maintained in the greenhouse (average seasonal temperature: 27.8 °C daytime/25.8 °C nighttime) until booting stage, and then, half of them were transferred into HNT growth chamber (30 °C daytime/28 °C nighttime, 14 h photoperiod) until harvest. Nip_vpp5 plants generally took 4–5 days longer to flower in both greenhouse (GH) and HNT conditions compared to the wildtype (Nip_WT), and average height of Nip_vpp5 plants at the seed filling stage was significantly lower than that of Nip_WT (Fig. S1a–c), but no significant effect of *vpp5* mutation on panicle number and panicle length was observed (Fig. S1d–e). However, SF was significantly improved. Although detrimental effect of HNT on SF was observed in both genotypes, Nip_vpp5 showed higher SF compared to Nip_WT (Fig. 1a**; **Fig. S2a). As observed in our previous study (Gann et al. 2023), Nip_vpp5 showed significantly lower (~ threefold lower) grain chalkiness compared to Nip_WT (Fig. 1b**; **Fig. S2b). Next, a negative effect of HNT on grain weight was observed in both genotypes, but significant differences between genotypes within a growing condition were not found (Fig. S3a). Finally, milled rice yield (MRY) and head rice yield (HRY) were significantly higher for Nip_vpp5 under HNT, but no significant difference was observed for grains ripened in the greenhouse (Fig. 1c–d). Interestingly, a small but significant increase in grain length was observed in Nip_vpp5 milled grains compared to Nip_WT (Fig. S3b). Overall, this analysis illuminated a positive effect of *vpp5* mutation on major yield components: SF, MRY, and HRY.

**Fig. 1:**
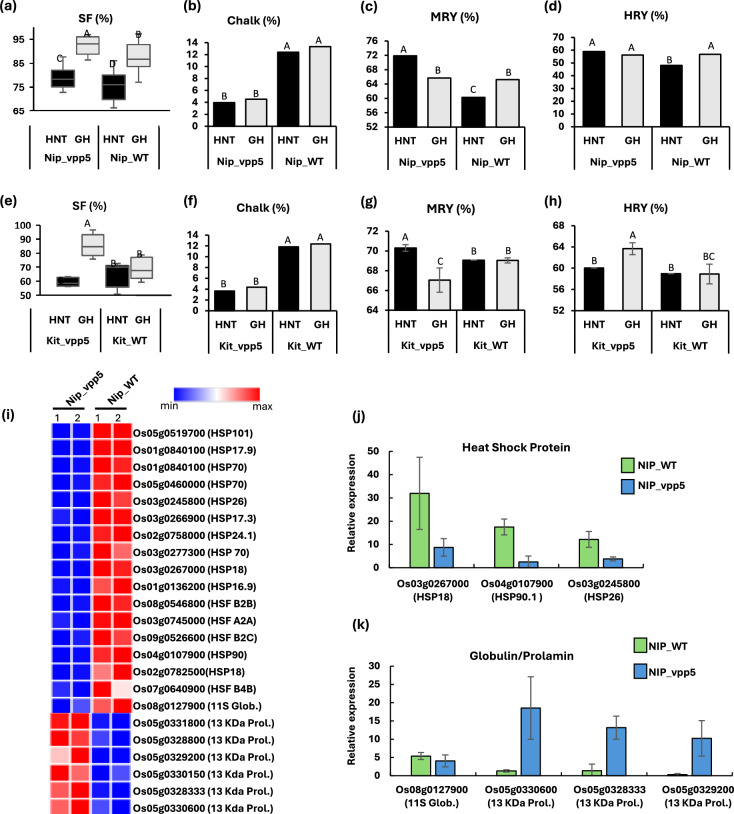
Grain quality and yield analysis of Nipponbare (Nip) or Kitaake (Kit) rice lines harboring vpp5 mutation: Nip_vpp5 **a**–**d** and Kit_vpp5 **e**–**f**. The experiment was conducted in two environments: greenhouse (GH) and growth chamber set at high nighttime temperature (HNT). **a**, **e** Spikelet fertility, **b**, **f** percent chalk per grain, **c**, **g** milled rice yield (MRY), and **d**, **h** head rice yield (HRY: number of unbroken kernels), (i) expression pattern of heat-shock protein (HSP) and heat stress transcription factor (HSF) genes in Nip_vpp5 and Nip_WT. Expression values were obtained from RNA-seq analysis using two biological replicates of each genotype, **j**–**k** gene expression analysis by real time qPCR on heat shock protein (HSP), 11S globulin (Glob.), and 13 KDa prolamin (Prol.) genes in the developing caryopses. Relative expression was calculated against rice ubiquitin 2 gene. Primers are shown in Fig. S5e. Error bars in **a**, **e** show standard deviation of data from 15 plants in each genotype/environment. Percent chalk and milling yield were determined on 3 replicates of 100 grains each on WinSEEDLETM 2024 and Zavvaria PAZ-1DTA mill, respectively. Significance in Tukey’s multiple comparison at p<0.05 is shown by capital letters. Error bars in **j**–**k** show standard derivation based on 2–4 replicates

Next, we determined the effect of *vpp5* mutation in rice cv. Kitaake. Using the CRISPR/Cas9 vector and transformation methods described earlier (Gann et al. 2023), we developed two *vpp5* mutants in Kitaake (Kit_vpp5-6 and Kit_vpp5-7). Kit_vpp5-6 contained a biallelic homozygous insertion-deletion consisting of 256 bp deletion and 13 bp insertion, and Kit_ vpp5-7 contained biallelic heterozygous 240 bp deletion with or without 2 bp insertion between the targeted sites (Fig. S4a–b). Gene expression analysis by qPCR showed that *VPP5* was downregulated in the caryopses of both lines; however, a significant difference (*p* < 0.01) was observed only in Kit_vpp5-6 (Fig. S4c). Based on this, Kit_vpp5-6 was selected for the yield analysis. First generation seeds (T1) of Kit_vpp5-6, referred to as Kit_vpp5, hereafter, were sown in the greenhouse and the plants at booting stage were treated with HNT as described above. Notably, Kit_vpp5 showed improved SF in the greenhouse and reduced chalkiness under HNT compared to the wildtype (Kit_WT) (Fig. 1e–f), indicating a positive effect of *vpp5* mutation on these traits. This is highly significant as it validates the effect of *vpp5* mutation on two major yield traits. Finally, MRY and HRY in Kit_vpp5 were variable but significantly higher than that of Kit_WT in at least one environment (Fig. 1g–h). Overall, the positive effect of *vpp5* on grain quality and yield was observed in rice varieties, which are generally non-chalky, but succumb to chalkiness under HNT.

Previous studies suggested that V-PPase affects starch-related processes (Li et al. 2014; Gann et al. 2023). Corroborating with that, Nip_vpp5 showed a slower rate of post-germinative growth that correlated with delayed starch hydrolysis (Gann et al. 2025). Here, we carried out RNA-seq analysis in developing caryopses to understand transcriptional changes associated with improved grain quality in Nip_vpp5. We selected caryopses at 10 days after flowering (10DAF) as *VPP5* is highly expressed at this stage (Gann et al. 2023). Principal component analysis showed > 50% variance in the transcriptional profile of Nip_vpp5 and Nip_WT, and differential expression analysis found 615 differentially expressed genes (FDR < 0.05, |log2FC|> 1) (Fig. S5a–b). As expected, the key starch pathway genes were differentially expressed (Fig. S5c); however, gene enrichment analysis showed that heat response processes were downregulated. Specifically, gene ontology (GO) terms, ‘response to temperature stimulus’, ‘response to heat’, and ‘protein folding’ were highly significant (FDR < 0.01) (Fig. S5d). These pathways include heat shock protein (HSP) and heat shock transcription factor (HSF), 16 of which were downregulated (FDR < 0.05, |log2FC|> 1) in Nip_vpp5 (Fig. 1i). We validated these data by qPCR on a subset of these genes (Fig. 1j). HSPs and HSF play a major role in heat stress response, but they also exert developmental controls in non-stress condition, *e.g.*, small HSP (sHSP) accumulate in embryo and endosperm during seed development (Waters and Vierling 2020). Further, since heat induces *HSP* expression in rapidly growing cells and heat is also a major inducer of grain chalkiness in most cultivars, a correlation of HSP expression and chalkiness is logical. This hypothesis is supported by studies that showed upregulation of HSP and HSF in rice caryopses under elevated temperature and accumulation of small HSP in chalky kernels (Yamakawa et al. 2007; Yamakawa and Hakata 2010). Thus, downregulation of sHSP genes in Nip_vpp5 arguably contributes to reduced chalkiness. Next, we found that a set of 13 kDa prolamin genes was upregulated in Nip_vpp5, while 11S globulin was downregulated (Fig. 1i, k). Lower abundance of 13 kDa prolamins and higher abundance of 11S globulin have been linked with chalky appearance of the grains (Lin et al. 2017; Yamakawa et al. 2007; Yamakawa and Hakata 2010). Prolamin is the major component of protein bodies in rice endosperm, and its deficiency could impact their morphology. Disturbance in protein body number and morphology was pointed out by Li et al. as the basis of chalky endosperm. In conclusion, this study showed that CRISPR-based modulation of *VPP5* generated a reliable allele for improved SF and reduced chalkiness under HNT. The underlying mechanism involves a range of metabolic changes that are not limited to starch biosynthesis and include differential accumulation of prolamin and sHSP.

## Supplementary Information

Below is the link to the electronic supplementary material.

Supplementary file1 (PPTX 5477 KB)

## Data Availability

Please contact authors for the availability of additional data on differential gene expression.
