# Supplementary material for: CRISPR/Cas9-based modulation of V-PPase expression in rice improves grain quality and yield under high nighttime temperature
Source: Plant Cell Rep. 2025 May 10;44(6):119. doi: 10.1007/s00299-025-03504-y (PMC12065718; doi:10.1007/s00299-025-03504-y)
Supplement: Supplementary file 1 — Supplementary file1 (PPTX 5477 KB) [file 299_2025_3504_MOESM1_ESM.pptx]

## Slide 1
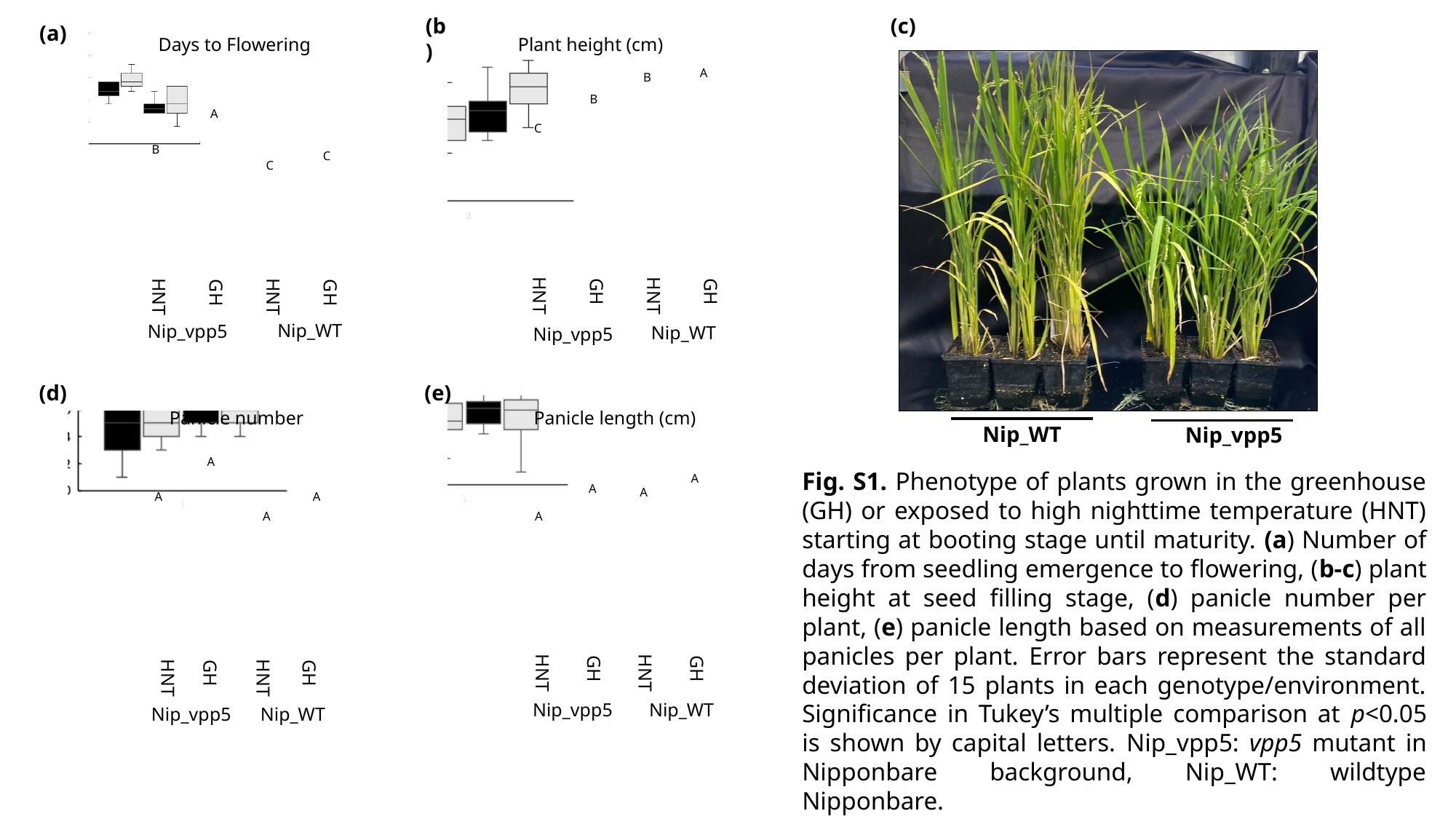

(b)
(c)
(a)
Days to Flowering
Plant height (cm)
A
B
C
Nip_WT
Nip_vpp5
A
B
B
C
C
GH
GH
GH
GH
HNT
HNT
HNT
HNT
Nip_WT
Nip_vpp5
Nip_WT
Nip_vpp5
(d)
(e)
Panicle number
Panicle length (cm)
A
Fig. S1. Phenotype of plants grown in the greenhouse (GH) or exposed to high nighttime temperature (HNT) starting at booting stage until maturity. (a) Number of days from seedling emergence to flowering, (b-c) plant height at seed filling stage, (d) panicle number per plant, (e) panicle length based on measurements of all panicles per plant. Error bars represent the standard deviation of 15 plants in each genotype/environment. Significance in Tukey’s multiple comparison at p<0.05 is shown by capital letters. Nip_vpp5: vpp5 mutant in Nipponbare background, Nip_WT: wildtype Nipponbare.
A
A
A
A
A
A
A
GH
GH
HNT
HNT
GH
GH
HNT
HNT
Nip_vpp5
Nip_WT
Nip_vpp5
Nip_WT

## Slide 2
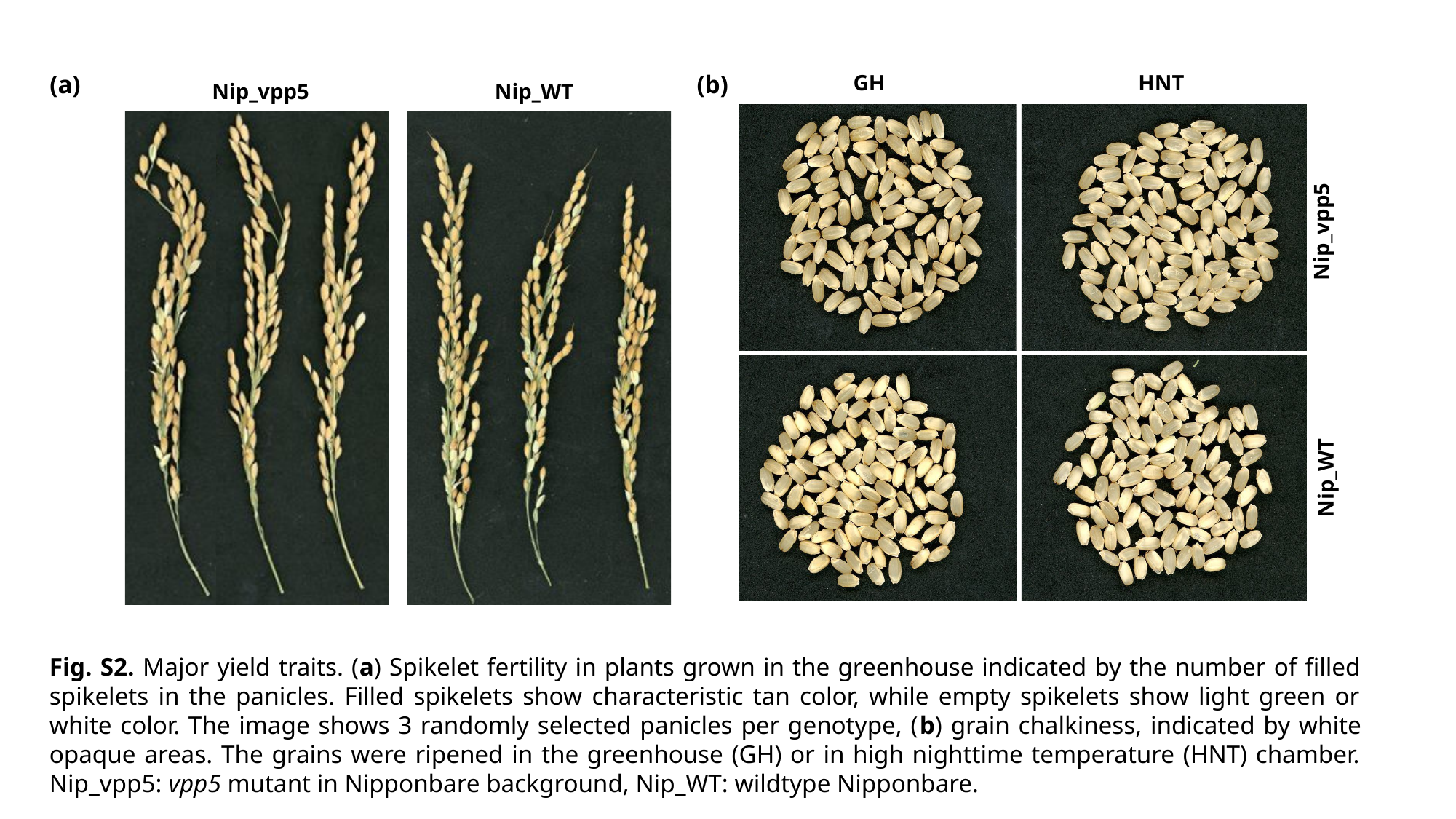

HNT
(a)
(b)
GH
Nip_WT
Nip_vpp5
Nip_vpp5
Nip_WT
Fig. S2. Major yield traits. (a) Spikelet fertility in plants grown in the greenhouse indicated by the number of filled spikelets in the panicles. Filled spikelets show characteristic tan color, while empty spikelets show light green or white color. The image shows 3 randomly selected panicles per genotype, (b) grain chalkiness, indicated by white opaque areas. The grains were ripened in the greenhouse (GH) or in high nighttime temperature (HNT) chamber. Nip_vpp5: vpp5 mutant in Nipponbare background, Nip_WT: wildtype Nipponbare.

## Slide 3
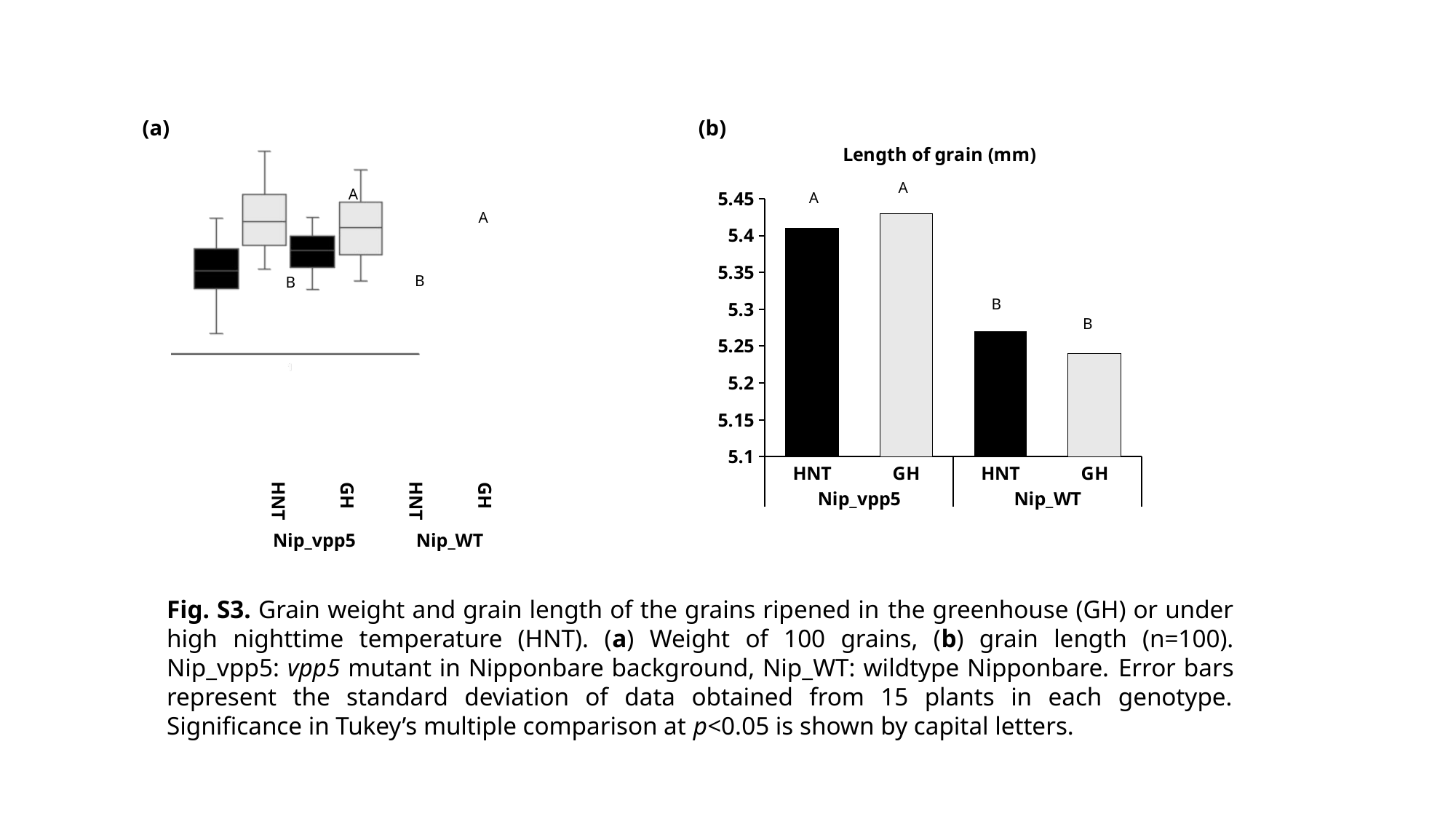

(a)
(b)
### Chart: Length of grain (mm)
| Category | |
|---|---|
| HNT | 5.41 |
| GH | 5.43 |
| HNT | 5.27 |
| GH | 5.24 |A
A
A
A
B
B
B
B
GH
GH
HNT
HNT
Nip_vpp5
Nip_WT
Fig. S3. Grain weight and grain length of the grains ripened in the greenhouse (GH) or under high nighttime temperature (HNT). (a) Weight of 100 grains, (b) grain length (n=100). Nip_vpp5: vpp5 mutant in Nipponbare background, Nip_WT: wildtype Nipponbare. Error bars represent the standard deviation of data obtained from 15 plants in each genotype. Significance in Tukey’s multiple comparison at p<0.05 is shown by capital letters.

## Slide 4
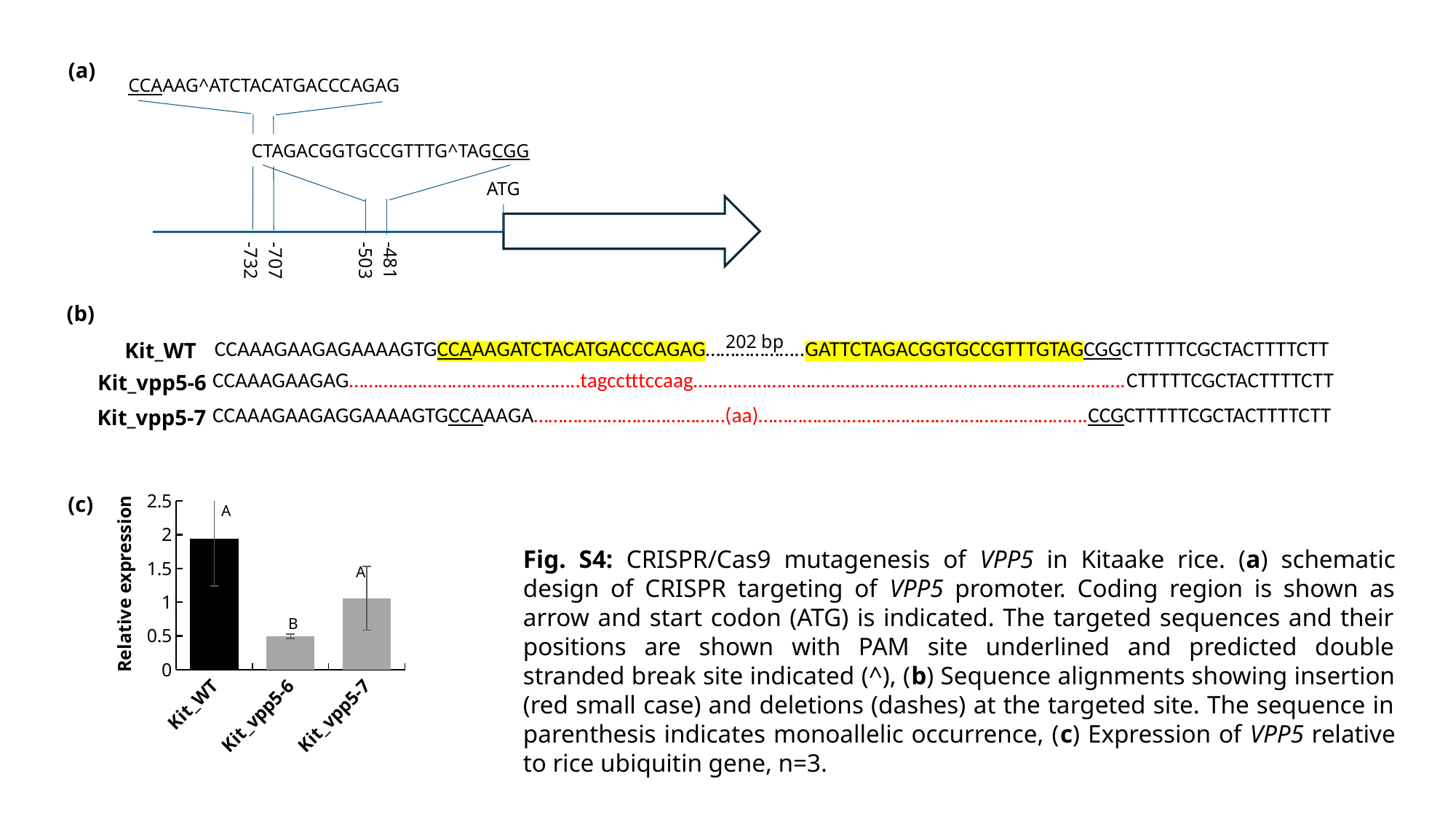

(a)
CCAAAG^ATCTACATGACCCAGAG
CTAGACGGTGCCGTTTG^TAGCGG
ATG
-732
-707
-503
-481
(b)
202 bp
CCAAAGAAGAGAAAAGTGCCAAAGATCTACATGACCCAGAG………………..GATTCTAGACGGTGCCGTTTGTAGCGGCTTTTTCGCTACTTTTCTT
Kit_WT
CCAAAGAAGAG………………………………………..tagcctttccaag…………………………………………………………………………….CTTTTTCGCTACTTTTCTT
Kit_vpp5-6
CCAAAGAAGAGGAAAAGTGCCAAAGA…………………………………(aa)………………………………………………………….CCGCTTTTTCGCTACTTTTCTT
Kit_vpp5-7
### Chart
| Category | |
|---|---|
| Kit_WT | 1.9421871622317202 |
| Kit_vpp5-6 | 0.4964260922201947 |
| Kit_vpp5-7 | 1.0600661521663346 |(c)
A
Fig. S4: CRISPR/Cas9 mutagenesis of VPP5 in Kitaake rice. (a) schematic design of CRISPR targeting of VPP5 promoter. Coding region is shown as arrow and start codon (ATG) is indicated. The targeted sequences and their positions are shown with PAM site underlined and predicted double stranded break site indicated (^), (b) Sequence alignments showing insertion (red small case) and deletions (dashes) at the targeted site. The sequence in parenthesis indicates monoallelic occurrence, (c) Expression of VPP5 relative to rice ubiquitin gene, n=3.
A
Relative expression
B

## Slide 5
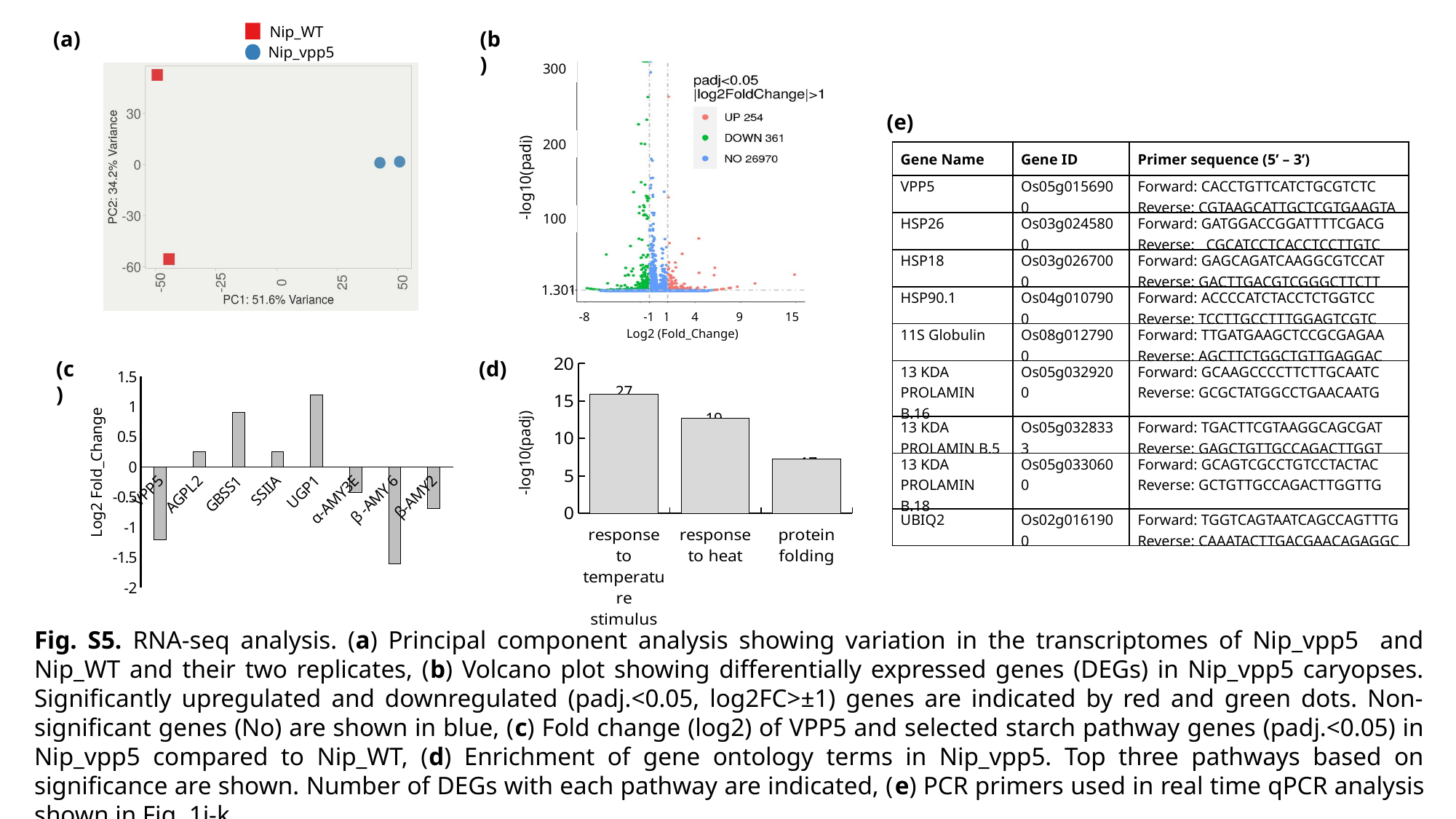

Nip_WT
Nip_vpp5
(a)
(b)
300
(e)
200
| Gene Name | Gene ID | Primer sequence (5’ – 3’) |
| --- | --- | --- |
| VPP5 | Os05g0156900 | Forward: CACCTGTTCATCTGCGTCTC Reverse: CGTAAGCATTGCTCGTGAAGTA |
| HSP26 | Os03g0245800 | Forward: GATGGACCGGATTTTCGACG Reverse: CGCATCCTCACCTCCTTGTC |
| HSP18 | Os03g0267000 | Forward: GAGCAGATCAAGGCGTCCAT Reverse: GACTTGACGTCGGGCTTCTT |
| HSP90.1 | Os04g0107900 | Forward: ACCCCATCTACCTCTGGTCC Reverse: TCCTTGCCTTTGGAGTCGTC |
| 11S Globulin | Os08g0127900 | Forward: TTGATGAAGCTCCGCGAGAA Reverse: AGCTTCTGGCTGTTGAGGAC |
| 13 KDA PROLAMIN B.16 | Os05g0329200 | Forward: GCAAGCCCCTTCTTGCAATC Reverse: GCGCTATGGCCTGAACAATG |
| 13 KDA PROLAMIN B.5 | Os05g0328333 | Forward: TGACTTCGTAAGGCAGCGAT Reverse: GAGCTGTTGCCAGACTTGGT |
| 13 KDA PROLAMIN B.18 | Os05g0330600 | Forward: GCAGTCGCCTGTCCTACTAC Reverse: GCTGTTGCCAGACTTGGTTG |
| UBIQ2 | Os02g0161900 | Forward: TGGTCAGTAATCAGCCAGTTTG Reverse: CAAATACTTGACGAACAGAGGC |
-log10(padj)
100
1.301
### Chart
| Category | |
|---|---|
| response to temperature stimulus | 15.91427377312982 |
| response to heat | 12.723480200963758 |
| protein folding | 7.2475578179650375 |-8
-2
-1
1
4
9
15
-8
-1
1
4
9
15
Log2 (Fold_Change)
(c)
(d)
### Chart
| Category | |
|---|---|
| VPP5 | -1.2 |
| AGPL2 | 0.25 |
| GBSS1 | 0.9 |
| SSIIA | 0.25 |
| UGP1 | 1.2 |
| α-AMY3E | -0.42 |
| β -AMY 6 | -1.6 |
| β-AMY2 | -0.69 |Log2 Fold_Change
27
19
-log10(padj)
17
Fig. S5. RNA-seq analysis. (a) Principal component analysis showing variation in the transcriptomes of Nip_vpp5 and Nip_WT and their two replicates, (b) Volcano plot showing differentially expressed genes (DEGs) in Nip_vpp5 caryopses. Significantly upregulated and downregulated (padj.<0.05, log2FC>±1) genes are indicated by red and green dots. Non-significant genes (No) are shown in blue, (c) Fold change (log2) of VPP5 and selected starch pathway genes (padj.<0.05) in Nip_vpp5 compared to Nip_WT, (d) Enrichment of gene ontology terms in Nip_vpp5. Top three pathways based on significance are shown. Number of DEGs with each pathway are indicated, (e) PCR primers used in real time qPCR analysis shown in Fig. 1j-k.
